# Post-Transplant Hypertension in Kidney Recipients: Current Knowledge, Gaps and Future Directions

**DOI:** 10.3390/jcm15124808

**Published:** 2026-06-21

**Authors:** Alicja Danieluk, Tomasz Pilecki, Bartosz Rutka, Krzysztof Mucha

**Affiliations:** 1 Department of Transplantology, Immunology, Nephrology and Internal Diseases, Medical University of Warsaw, 02-006 Warsaw, Poland; 2 Institute of Biochemistry and Biophysics, Polish Academy of Sciences, 02-106 Warsaw, Poland

**Keywords:** immunosuppression, kidney transplantation, post-transplant hypertension, resistant hypertension, therapeutic strategies

## Abstract

Cardiovascular disease remains the leading cause of mortality in kidney transplant recipients (KTRs). Arterial hypertension is present in a vast majority of patients after kidney transplantation, constituting the most prevalent cardiovascular comorbidity, and is a significant modifiable risk factor for other cardiovascular complications and graft loss. The 2024 Kidney Disease: Improving Global Outcomes (KDIGO) guidelines do not address blood pressure control strategies in KTRs, and the prior 2021 KDIGO recommendations targeting values below 130/80 mmHg rely primarily on data extrapolated from non-KTR populations. This represents an existing evidence gap in the management of post-transplant hypertension. Dihydropyridine calcium channel blockers and angiotensin receptor blockers remain first-line antihypertensive medications, although most studies assessing their effectiveness in KTRs date back more than 15 years. The current treatment guidelines are based largely on limited and outdated data. Optimal selection and individualization of immunosuppressive therapy and—when feasible—its modification in some KTRs may be important in improving blood pressure control. This includes, for example, a reduction in the calcineurin inhibitor or steroid dose, as well as the use of mTOR inhibitors or belatacept. The lack of large, up-to-date randomized trials in the KTR population underscores the pressing need for further extensive research focused on this patient group.

## 1. Introduction

Kidney transplantation (KTx) is the optimal treatment method for end-stage kidney disease, improving survival and quality of life compared to dialysis [1,2,3,4,5,6]. However, kidney transplant recipients (KTRs) are characterized by significantly higher cardiovascular risk compared to the general population. It is known that cardiovascular diseases (CVDs) remain the primary cause of morbidity and mortality following KT [1,2,3,4,5]. Arterial hypertension, the most prevalent cardiovascular comorbidity, affects up to 70–95% of KTRs and is a major contributor to graft dysfunction [1,2,3,4]. The management of arterial hypertension in KTRs requires a comprehensive and individualized approach, possibly differing from those used in the general population. The pathophysiology of arterial hypertension following KTx is extremely complex, due to its multifactorial and heterogeneous nature. It typically arises from the overlap of risk factors such as age, sex, body weight, vascular stiffness, diabetes or dyslipidemia with factors closely related to the transplantation process itself [1,4]. The predominant pathomechanisms vary among individual patients, and may depend on graft function, immunosuppression, renal denervation, vascular complications such as transplant renal artery stenosis (TRAS), the individual’s immune profile, donor-related factors and/or the patient’s prior treatment history [1,2,3,4,5,7,8,9,10]. Despite intensive pharmacotherapy targeting various pathophysiological mechanisms, a significant proportion of patients develop resistant hypertension, requiring combination therapy with various antihypertensive drug classes and failing to achieve recommended blood pressure (BP) targets [1,4,6,11].

The purpose of this review is to present the state of knowledge regarding arterial hypertension and patient management following KTx, in order to better understand post-KTx hypertension and develop more effective treatments to improve the quality of life of these patients.

This review was based on a comprehensive literature search conducted across the PubMed and Cochrane Library databases, as well as an analysis of clinical practice guidelines, including KDIGO. The selection focused on English-language publications regarding arterial hypertension management in KTRs. Articles and guidelines published up to 26 May 2026 were included.

## 2. Blood Pressure Targets

Currently, the KDIGO guidelines are the principal recommendations for the management of KTRs in clinical practice. The latest guidelines for arterial hypertension treatment, including the KTR population, were published in 2021 [7,8]. Given the lack of references for KTRs in the latest KDIGO 2024 chronic kidney disease (CKD) guidelines, the prior guidelines remain the primary source of information on the treatment of the disease in this group [7,8,12].

The KDIGO 2021 recommendation for treatment of arterial hypertension in KTRs is to keep the BP below 130/80 mmHg [7,8]. However, they underline the fact that this target was upheld from the 2012 KDIGO guidelines, due to a lack of updated randomized trials examining different BP targets in KTRs in terms of important clinical outcomes such as mortality or graft survival. Although KDIGO 2021 recommends a target systolic blood pressure (SBP) below 120 mmHg for the CKD population, a deliberate decision not to apply such an intensive BP target to KTRs was made [7,8]. These recommendations were influenced by the SPRINT study results published in 2015, which revealed that intensive antihypertensive therapy aimed at achieving SBP < 120 mmHg was associated not only with additional cardiovascular and mortality benefits, but also an increased risk of acute kidney injury (AKI) and a higher rate of transient hemodynamically mediated estimated glomerular filtration rate (eGFR) decline compared to less-intensive approaches. However, as KTRs were not included in the SPRINT study, these conclusions must be interpreted with caution. It is necessary to acknowledge that these findings represent an extrapolation of data, rather than direct evidence in the KTR population [7,8,13].

The KDIGO guidelines also emphasize the need for an individualized approach in the treatment of arterial hypertension, taking the CVD risk profile, transplanted kidney function and risk of adverse events into account. It has been noted that more intensive therapy and lower target values may be considered in certain cases, provided that the patients cooperate fully with the treatment and that their clinical status is closely monitored [3,4,7,8].

The Collaborative Transplant Study (CTS) showed the importance of determining adequate BP targets for long-term kidney function and patient survival. A retrospective analysis of 62,556 adult KTRs from 209 centers in 39 countries assessed the impacts of different BP targets on long-term graft survival and patient mortality. At 1 year after KTx, 77% of KTRs had hypertension. Stage 1 hypertension (130–139/80–89 mmHg) was associated with an 11% increase in the risk of graft loss. Stage 2 hypertension (≥140/≥90 mmHg) was linked to a 55% increase in risk and was also associated with higher mortality. There were no differences between patients with BP < 120/80 mmHg and those with 120–129/<80 mmHg, leaving the benefits of targeting a BP below 120 mmHg unclear. However, it must be noted that the analysis was based on a single measurement taken in a physician’s office a year after KTx, with no measurement standardization, potentially affecting the accuracy and reliability of these data [14]. Ambulatory Blood Pressure Monitoring (ABPM) is considered a better diagnostic method for arterial hypertension and follow-up [15,16,17]. Pisano et al. [11] conducted a meta-analysis and found that ABPM was more effective for the diagnosis of uncontrolled hypertension in KTRs than office BP (56% vs. 47%); this result is particularly relevant given the high prevalence of nocturnal hypertension and blood pressure profile disturbances in the KTR population.

## 3. Pharmacological Treatment

### 3.1. Recommended First-Line Therapy

Antihypertensive treatment in the overall population is generally based on a combination therapy consisting primarily of angiotensin-converting enzyme inhibitors (ACEIs), angiotensin II receptor blockers (ARBs), calcium channel blockers (CCBs) and thiazide-like diuretics [15,16,17]. However, the 2021 KDIGO guidelines recommend a partly different therapeutic approach for KTRs, considering factors specific to this group and the importance of prioritizing the prevention of graft loss [7,8,18,19].

According to data presented in the 2024 Cochrane review and 2021 KDIGO guidelines, dihydropyridine (DHP) CCBs and ARBs are established as the first-line antihypertensive therapies in KTRs. Meanwhile, data on the use of ACEIs are associated with lower confidence, deviating from the guidelines for the non-KTR population. However, ACEIs may be beneficial in some groups of KTRs, such as patients with cardiovascular comorbidities and proteinuria. Diuretics are likewise not used as first-line agents for BP control. Nevertheless, they may be used in some KTRs, especially in cases of concomitant volume overload in the early post-transplant period and in patients with heart failure or reduced kidney function. Other antihypertensives, such as beta-blockers, alpha-1 blockers, centrally acting agents and mineralocorticoid receptor antagonists, may also be considered in selected KTRs with resistant hypertension. However, evidence supporting their use in this population remains limited, and the KDIGO 2021 evidence review found no significant effects on graft loss, cardiovascular events and all-cause mortality [4,6,7,8].

It is important to note that the majority of studies included in the Cochrane 2024 meta-analysis date back more than a decade, with several featuring wide confidence intervals, reflecting substantial statistical uncertainty (detailed characteristics and results are summarized in Table 1) [6]. Significant progress in transplantation over the years has led to substantial changes in the characteristics of KTRs, particularly regarding their more advanced age, the prevalence of comorbidities and immunosuppressive regimens [12]. This imposes a serious limitation on any attempt to directly apply the results of the meta-analysis to the current KTR population.

#### 3.1.1. Calcium Channel Blockers

This class is divided, according to their chemical structure, into dihydropyridines (e.g., nifedipine, nitrendipine, amlodipine, lacipidine, lercanidipine, nicardipine), benzothiazepines (e.g., diltiazem) and phenylalkylamines (e.g., verapamil). The primary mechanism behind the antihypertensive effect of CCBs is the inhibition of calcium influx through voltage-dependent L-type (long-lasting) calcium channels during membrane depolarization of vascular smooth muscle cells (VSMCs). This results in reduced intracellular calcium levels and subsequent vasodilation of small resistance arteries.

DHP CCBs have vascular selectivity; that is, they show preference for VSMC calcium channels rather than cardiomyocyte channels. When acutely administered, DHP CCBs reduce total peripheral resistance and mean blood pressure and may lead to a reflex increase in cardiac output due to sympathetic activation. After chronic usage, cardiac output returns toward pretreatment levels, while mean arterial pressure and systemic vascular resistance remain reduced. These changes are associated with a relaxation of large arteries and, thus, with reduced arterial stiffness, causing decreases in central pressure and pulse pressure. In contrast, non-DHP have cardiac selectivity; i.e., they act more effectively in cardiomyocytes, decreasing heart rate, intracardiac conduction and myocardial contractility. Moreover, CCBs dilate afferent arterioles in the glomeruli. In the KTR population, they can reverse the renal vasoconstriction triggered by calcineurin inhibitors (CNIs), thus protecting kidney graft function [1,2,4,6,20].

The Cochrane 2024 meta-analysis revealed that CCB-based therapy leads to a significant reduction in graft loss when compared to placebo or standard care alone (24 studies, 3577 participants; RR: 0.84, 95% CI: 0.75 to 0.95, I2 = 0%). Furthermore, CCBs were associated with lower all-cause mortality (23 studies, 3327 participants; RR: 0.83, 95% CI: 0.72 to 0.95, I2 = 0%). However, this evidence is only of moderate certainty [6].

The effects of CCBs on renal function, measured in terms of estimated glomerular filtration rate (eGFR), vary between available studies. A 2024 Cochrane meta-analysis of 11 studies with 2250 participants found little or no difference in eGFR (MD: 1.89 mL/min/1.73 m^2^, 95% CI: −0.70 to 4.48, I2 = 48%), although this evidence is of low certainty. After conducting a subgroup analysis, it was found that such heterogeneity was partly associated with the different CCB classes used in the studies analyzed. DHP CCBs showed a stronger, statistically significant increase in eGFR (MD: 4.92 mL/min/1.73 m^2^, 95% CI: 0.46 to 9.39, I2 = 43%), while non-DHP drugs did not have a significant effect on eGFR improvement (MD: −0.37 mL/min/1.73 m^2^, 95% CI: −2.20 to 1.46, I2 = 5%) [6].

CCBs, particularly DHP-type, are considered to be first-line agents in KTRs. The data suggest that, among the antihypertensive drugs, they appear to have the most well-documented potential beneficial effects on both graft and overall patient survival. However, it is important to stress typical adverse effects such as edema or proteinuria, and potential interactions with CNIs, thus requiring drug concentration monitoring and dose adjustment [4,6,7,21].

#### 3.1.2. Angiotensin Receptor Blockers

ARBs work by competitively binding to AT1 receptors, thus inhibiting the RAS axis. Through blocking the activity of angiotensin II, they lead to vasodilation and a decline in BP, as well as a decrease in intraglomerular pressure, resulting in reduced proteinuria [4,6,7].

Although a 2024 Cochrane review of ARBs showed little or no differences in all-cause mortality and eGFR, a potentially relevant impact on graft survival in patients treated with ARBs was found. In particular, in 6 studies involving 892 participants, the use of ARBs was associated with a substantially lower risk of graft loss (RR: 0.35; 95% CI: 0.15 to 0.84, I2 = 0%); however, the certainty of this evidence was rated as low. Due to the limited number of available studies, small study groups, as well as heterogeneity and a high risk of bias, these results should be interpreted with caution [6,7].

While ARBs are supported by evidence of low certainty, they have potential protective effects on graft function, especially in patients with proteinuria. Despite having some adverse effects (e.g., anemia, hyperkalemia, risk of acute decline in graft function; in some cases, even fulfilling the criteria for AKI), they are recommended as first-line therapeutics alongside CCBs [7,8]. However, it is important to mention the greater risk in patients treated with CNIs or with suboptimal graft function, especially in the early post-transplant period.

#### 3.1.3. Angiotensin-Converting Enzyme Inhibitors

ACEIs, similarly to ARBs, inhibit the RAS pathway, leading to vasodilation and, subsequently, to decreases in BP and proteinuria [4,6,7]. Data for ACEIs evaluated in the Cochrane 2024 review (6 studies, 718 participants) revealed that ACEIs may make little or no difference in terms of graft loss (RR: 0.75; 95% CI: 0.49–1.13, I2 = 0%; low certainty of evidence). Similarly, unclear conclusions were obtained in an assessment of the incidence of AKI in the context of ACEI treatment and the potential loss of a sole transplanted kidney (very low certainty evidence, 2 studies, 82 participants). Therefore, it is not possible to provide a reliable evaluation of their use in KTRs [6].

In clinical practice, concerns related to the risk of deterioration of glomerular filtration in KTRs during ACEI therapy are largely based on pathophysiological assumptions regarding RAS inhibition, rather than on solid data obtained from randomized trials. Given the lower incidence of graft loss in ARB groups, the 2021 KDIGO guidelines do not recommend the use of ACEIs as a first-line treatment in KTRs, favoring ARBs. However, ACEIs may be considered in some KTRs, such as those with proteinuria, chronic graft dysfunction, high cardiovascular risk or diabetes [4,6,7,8].

### 3.2. Modification of Immunosuppressive Therapy in the Management of Arterial Hypertension

Conversion, reduction or withdrawal of immunosuppressive therapy might be an additional strategy for treating resistant hypertension in some KTRs. However, given the importance of proper immunosuppression, care should be taken when modifying immunosuppressive regimens for the purpose of improving BP control in KTRs. Any decision regarding treatment regimen changes should take the individual’s immunological status and potential cardiovascular benefits into consideration, as well as any implications for kidney transplant function [1,2,4,9].

#### 3.2.1. Calcineurin Inhibitor Conversion, Reduction or Withdrawal

Calcineurin inhibitors (CNIs) might contribute to the development of post-transplant hypertension. This effect is mediated mostly through enhanced constriction, impaired vasodilation, RAS activation and increased sodium retention. Consequently, reducing the CNI dose may help to decrease nephrotoxicity and improve BP control in this group of patients [1,4,7,22]. Meier et al. [23] assessed cyclosporine-to-tacrolimus conversion in patients with chronic allograft nephropathy and observed improved graft function as well as lower BP, which was achieved by reducing the intensity of antihypertensive therapy. Mourer et al. [24] randomized 119 KTRs receiving a triple therapy regimen including steroids, CNI and mycophenolate mofetil (MMF) to either CNI withdrawal with continuation of MMF or MMF withdrawal with continuation of concentration-controlled CNI therapy. It was shown that CNI withdrawal resulted in a significant decrease in both daytime and nighttime BP. Daytime SBP was reduced by 1.6 mmHg/year (*p* = 0.018) and nighttime SBP by 1.9 mmHg/year (*p* = 0.008), while diastolic blood pressure (DBP) decreased by 1.3 mmHg/year (*p* = 0.002) during the day and by 1.3 mmHg/year (*p* = 0.014) at night. Discontinuation of MMF did not result in a significant decrease. Wong et al. [25] assessed the effects of a 50% reduction in the cyclosporine (CsA) dose on BP and uric acid levels in 31 KTRs over a 6-month follow-up period after CsA reduction. A significant decrease in SBP from 132 ± 16 to 125 ± 15 mmHg (*p* = 0.014) was observed after 6 months in individuals receiving decreased CsA doses, along with a reduction in the number of antihypertensive agents required. Thervet et al. [26] assessed the efficacy and safety of discontinuing CsA therapy after conversion from azathioprine (AZA) to MMF among KTRs. A significant reduction in SBP was observed as the average number of antihypertensive medications taken decreased (1.29 ± 1.2 initially, compared to 0.59 ± 0.8 upon the last visit; *p* = 0.006). However, it is worth noting that histologic deterioration was documented in 50% of these patients. A multicenter randomized pilot study involving 87 low-risk adult KTRs was conducted by Grinyo et al. [27] to evaluate the potential for early tacrolimus (TAC) withdrawal. Two treatment regimens based on both sirolimus and TAC were compared, and the results showed a significant reduction in DBP in the group where TAC was withdrawn (80.4 vs. 75.6 mmHg, *p* = 0.03), while the rate of rejection remained comparable (10.3% vs. 11.1% after protocol amendment).

#### 3.2.2. Steroid Avoidance or Withdrawal

Evidence on the role of steroid withdrawal or reduction in improving BP control in KTRs remains limited and inconsistent. Pascual J et al. [28] conducted an analysis on steroid avoidance or withdrawal in KTRs (30 randomized controlled studies, 5949 patients). The effects on blood pressure were rarely reported, limited to a reduction in the use of antihypertensives in 3 steroid withdrawal studies. The avoidance or withdrawal of steroids was not associated with increased mortality or graft loss, despite an increase in acute rejection. A systematic review on very early steroid withdrawal (within a few days after KTx) or complete avoidance in patients treated with antibody induction and CNI with MMF (9 studies, 1934 participants) showed no significant differences in mean BP, serum creatinine, creatinine clearance and acute rejection [29]. The recent 2026 Cochrane review on steroid avoidance or withdrawal compared to maintenance reported no significant difference in patient death or graft loss up to 1 year after transplantation; however, steroid avoidance was associated with reduction in patient death at 5 years. The risk of biopsy-proven acute rejection in steroid withdrawal regimens was similar to that under the steroid maintenance approach. No differences in cardiovascular events were found, but the BP was not analyzed. Nevertheless, the authors stressed that the certainty of evidence was low [30].

#### 3.2.3. Conversion of Immunosuppression

Studies have drawn attention to the possibility of changing the regimen from CNIs to mTOR inhibitors (also known as PSIs) or belatacept-based immunosuppression; however, this approach still requires further research [1,2,31,32,33]. Murbraech et al. [31] observed significantly decreased DBP (−8 mmHg, *p* = 0.002) in the everolimus group at 3 years after early (7-week) conversion from CsA to everolimus in KTRs. In a case–control study, Seibert et al. [32] compared the effects of belatacept and CsA treatment on peripheral and central BP in 46 KTRs. Although no significant differences were found in peripheral BP, a significantly lower central aortic augmentation pressure was observed in the belatacept group compared to the CsA group. Masson et al. [33] performed a meta-analysis and reported a number of clinical benefits of belatacept compared with CNIs in KTRs (5 studies, 1535 recipients). No differences were observed in the risk of kidney graft loss and return to dialysis up to 3 years post-transplant; however, kidney function (eGFR) in the belatacept group was significantly better than in the CNI group (4 studies, 1083 recipients; MD: 9.96 mL/min/1.73 m^2^, 95% CI: 3.28 to 16.64). Additionally, belatacept recipients had lower BP (2 studies, 658 recipients; SBP MD: −7.51 mmHg, 95% CI: −10.57 to −4.46; DBP MD: −3.07 mmHg, 95% CI: −4.83 to −1.31). Treatment with belatacept was also associated with an improved lipid profile and a reduction in the rate of new-onset post-transplant diabetes. However, further long-term research is needed to fully evaluate these findings in the kidney transplant population.

#### 3.2.4. Pharmacokinetic Interactions

An important consideration in antihypertensive therapy in KTRs is the potential interactions with immunosuppressive therapy. Several CCBs—especially verapamil, diltiazem and nicardipine—significantly affect cyclosporine metabolism by increasing its plasma level and decreasing the required dosage [21]. Therefore, due to inhibition of CYP3A4 activity, careful therapeutic drug monitoring and appropriate dose adjustment are essential in CNI-based immunosuppressive treatments in the context of post-transplant hypertension.

### 3.3. Non-Antihypertensive Medications

Emerging evidence suggests potential effects on transplanted kidney function and improvements in BP control in some studies involving flozines (SGLT2is) and glucagon-like peptide type 1 receptor agonists (GLP-1RAs). Sánchez Fructuoso et al. [34], in a multicenter study involving 339 KTRs with diabetes, showed that the use of an SGLT2i led to a significant reduction in BP, with mean decreases in SBP by 4.63 mmHg (95% CI, −6.73 to −2.52) and DBP by 2.24 mmHg (95% CI, −3.49 to −1.00). Baslé et al. [35], in a French multicenter study, reported a significant reduction in SBP compared to baseline values, regardless of whether the patients had diabetes. The SBP and DBP were both reduced by 4 mmHg after 3 months, and the effect remained at a level of 2.5 mmHg (SBP) and 3 mmHg (DBP) after 6 months. Based on a meta-analysis of the effect of an SGLT2i on BP in 9 studies (853 patients), Lee et al. [36] reported a significant reduction in the mean DBP of 2.08 mmHg (95% CI, –3.02 to –1.15); however, no statistically significant changes were observed for SBP. Moreover, in a pooled analysis of 5 studies involving 3448 patients, treatment with a GLP-1RA had no significant effect on SBP [36]. In a multicenter retrospective cohort study, Vigara et al. [37] evaluated 96 KTRs with diabetes who initiated treatment with a GLP-1RA. A total of 84 patients were followed for a minimum of 6 months, while 61 had a follow-up of 12 months. There were significant reductions in SBP (−7.5 mmHg, *p* = 0.013; −7.3 mmHg, *p* = 0.004), proteinuria, body weight, glycosylated hemoglobin, total cholesterol and LDL cholesterol after 6 and 12 months. Moreover, eGFR remained stable, with no change in the TAC dose or trough level. In contrast, a systematic review conducted by Bellos et al. [38] did not reveal any statistically significant effect of SGLT2is and GLP-1RAs on SBP in KTRs. These inconsistent results can be primarily attributed to significant statistical heterogeneity. Small study groups and baseline differences in patient characteristics, as well as the observational nature of most studies, limit the ability to identify clinically significant effects. Therefore, further randomized trials are needed to assess the impacts of SGLT2is and GLP-1RAs on BP in KTRs.

## 4. Discussion

DHP CCBs and ARBs remain the first-line antihypertensive agents for KTRs according to the KDIGO 2021 guidelines, as discussed earlier [6,7,8,18,19]. ACEIs, diuretics, beta-blockers, alpha-1 blockers, centrally acting agents and mineralocorticoid receptor antagonists may be useful in some KTRs, depending on their treatment priorities and comorbidities [4,6,7,8,12,21]. However, evidence on the use of these antihypertensive drug classes in KTR population is limited, with current findings mainly based on low-certainty data [7,8,12].

Modification of immunosuppressive therapy might be an additional strategy in treating resistant hypertension in carefully selected KTRs. Given the associated immunological risk and potential implications for graft survival, great care should be taken when considering such an approach [1,2,4,9]. Moreover, potential effects on graft function and BP control have been demonstrated in research involving SGLT2is and GLP-1RAs; however the evidence remains inconsistent and further trials are necessary [34,35,36,37,38].

Despite undeniable progress in transplantation, knowledge on the diagnosis and treatment of hypertension in KTRs remains incomplete and unreliable at present. The fact that the evidence base for present systematic reviews is outdated, generally relying on low-certainty data obtained over 15 years ago, is particularly noteworthy [6]. The analyzed patients were younger, had fewer comorbidities, and were treated with different immunosuppressive regimens. This leads to a potential for methodological bias, creating a significant risk of developing new recommendations based on incorrect assumptions. Given the lack of comprehensive randomized trials in KTRs, current BP target recommendations are largely based on extrapolation of data from the general population [6] and treatment guidelines are defined on the basis of limited research involving small study groups [7,8,12].

**Figure 1:**
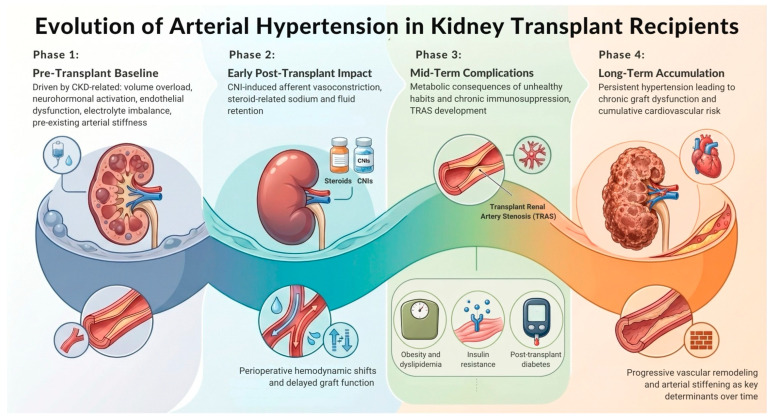
Evolution of arterial hypertension in kidney transplant recipients. Based on data compiled from [1,2,4,5,7,8,9,10].

Additionally, KTR population is highly heterogenous, characterized by numerous comorbidities and more complex pathophysiology of arterial hypertension (Figure 1). This has often led to the exclusion of this group from randomized trials, resulting in a lack of representative data for this group in new studies and forcing the extrapolation of recommendations applied to the general population of CKD patients [1,6,7,8,9].

We would like to point out that the current recommendations for treating hypertension in KTRs are missing comprehensive, personalized guidelines for specific patient groups that take into account their specific conditions. At present, KTRs are subject to the same rigid approach, regardless of their previous disease history. This underlines the need to address these specific factors in further clinical trials. Above all, post-transplant hypertension management should be individualized in terms of graft function, transplant timing, and recipient and donor characteristics (as shown in Table 2) [7,8].

We expect that large-scale, multicenter randomized trials in KTRs, designed with consideration of evidence from the general population and post-transplant registries, will provide clarity regarding optimal BP targets. Such studies should also evaluate novel treatment regimens across different subgroups, including graft function and immunosuppressive therapy, to better characterize both efficacy and safety outcomes.

While searching for breakthroughs in treatment of arterial hypertension in KTRs, it is important to consider whether we are making proper and full use of the tools we already have at our disposal. Non-pharmacological interventions are extremely important in this regard, as they lower BP and lead to reductions in CVD risk regarding both cardiovascular events and death [15,16,17]. Nevertheless, they should be adjusted for transplant-specific conditions, including CNI-associated salt-sensitive hypertension, tendency to hyperkalemia, post-transplant weight gain, intravascular and extravascular volume status and multiple comorbidities [1,2,4,5]. Consistent efforts to reduce body weight, cease smoking, limit alcohol consumption, increase physical activity and reduce sodium intake therefore play an important role in the treatment of these patients, and should not be overlooked. The Mediterranean or DASH diets can be recommended in some KTRs; however, kalemia monitoring is required [7,8,15,16,17]. Although these interventions may not completely replace pharmacotherapy, they increase its effectiveness and enable reductions in drug doses, as well as easier and safer achievement of set therapeutic goals [15,16,17].

A significant issue that requires closer evaluation is the actual compliance of patients after KTx in terms of following treatment recommendations. Pill overload is a major problem in this population, resulting from numerous comorbidities [7,9,12,39], which can lead to patients’ reluctance to adhere to recommendations, resulting in inadequate BP control and deterioration of graft function [7,12,39]. Such polytherapy increases the risk of pharmacological interactions which, in many cases, can be dangerous.

We found it important to mention studies describing the use of new and promising drugs targeting resistant hypertension in the general population, as they may be potential alternatives for the pharmacological management of arterial hypertension in KTRs. Aprocitentan, a dual endothelin receptor antagonist, was more effective at reducing nighttime BP than daytime BP in the general population [40]. Therefore, this more pronounced effect on nighttime BP may be beneficial for correction of nocturnal hypertension, which is frequent in KTRs. Aldosterone synthase inhibitors, such as baxdrostat and lorundrostat, exerted a strong antihypertensive effect in the general population [41,42,43]. Additionally, baxdrostat was effective in patients with eGFR <60 and ≥60 mL/min/1.73m^2^ [41]. Zilebesiran, a subcutaneously administered small interfering RNA that inhibits angiotensinogen synthesis, was effective as an antihypertensive add-on therapy in the general population [44,45]. Its subcutaneous administration and up to a 6-month duration of action may facilitate daily treatment routine and improve adherence to antihypertensive therapy in KTRs. However, these agents have not yet been tested in this population and require further research.

## 5. Conclusions and Future Directions

Available data on post-transplant hypertension management in KTRs largely date back more than 15 years and are based on low-certainty evidence from small, heterogeneous trials. The dynamic changes observed in this population over time (e.g., advanced age, higher prevalence of comorbidities and modified immunosuppressive regimens) make it difficult to apply existing recommendations to present-day patients. Furthermore, current guidelines on BP targets are primarily based on the extrapolation of data from other patient groups. The lack of up-to-date, large, randomized clinical trials addressing KTRs underlines the pressing need for new research on the diagnosis and treatment of arterial hypertension in this population. Targeted analyses of long-term transplant function and patient survival are particularly important, and the focus should remain on hard clinical outcomes, including graft survival, cardiovascular events, mortality and adverse events in the KTR population. The establishment of optimal therapeutic targets for this group, development of new BP measurement techniques, adjusted non-pharmacological approaches, tailored use of existing antihypertensive medications and, finally, the introduction of novel agents—once subjected to dedicated randomized trials—may contribute to more evidence-based and individualized care for KTRs in the future.

## Figures and Tables

**Table 1:** Antihypertensive treatment in KTRs: A summary of the results from the 2024 Cochrane meta-analysis [6].

Intervention Type Versus Placebo or Standard Care Alone (Control)	Outcome	No. of Studies	No. of Participants	Year(s) of Publication	Result (Effect Size) ^1^ Heterogeneity (I^2^), Certainty of Evidence
Calcium Channel Blockers (CCBs)	All-cause death	23	3327	1989–2013	RR 0.83 [0.72, 0.95] I2 = 0%; moderate certainty evidence
	Graft loss	24	3577	1986–2013	RR 0.84 [0.75, 0.95] I2 = 0%; moderate certainty evidence
	eGFR [mL/min/1.73 m^2^]	11	2250	1989–2011	MD 1.89 [−0.70, 4.48] I2 = 48%; low certainty evidence
	Acute kidney injury (AKI)	Not reported ^2^	Not reported ^2^	Not reported ^2^	Not reported ^2^
Angiotensin Receptor Blockers (ARBs)	All-cause death	6	1041	2004–2022	RR 0.69 [0.36, 1.31] I2 = 0%; low certainty evidence
	Graft loss	6	892	2004–2010	RR 0.35 [0.15, 0.84] I2 = 0%; low certainty evidence
	eGFR [mL/min/1.73 m^2^]	5	300	2005–2022	MD −1.91 [−6.20, 2.38] I2 = 57%; low certainty evidence
	Acute kidney injury (AKI)	1	66	2009	RR 4.71 [0.23, 94.58] I2 = not applicable; very low certainty evidence
Angiotensin-Converting Enzyme inhibitors (ACEIs)	All-cause death	7	702	1984–2015	RR 1.13 [0.58, 2.21] I2 = 0%; low certainty evidence
	Graft loss	6	718	1984–2015	RR 0.75 [0.49, 1.13] I2 = 0%; low certainty evidence
	eGFR [mL/min/1.73 m^2^]	4	509	1995–2015	MD −2.46 [−7.66, 2.73] I2 = 64%; low certainty evidence
	Acute kidney injury (AKI)	2	82	1995–2000	RR 10.63 [0.62, 183.77] I2 = not applicable; very low certainty evidence

^1^ RR = Risk Ratio (M-H, Random, 95% CI), MD = Mean Difference (IV, Random, 95% CI). ^2^ AKI was not reported in CCBs trials included in the 2024 Cochrane meta-analysis.

**Table 2:** Hypertension-related factors relevant to individualized blood pressure management [1,2,4,7,8,10].

Domain	Subcategory	Potential Impact
Graft function	eGFR ≥ 60 eGFR 30–59 eGFR 15–30	RAS stimulation, volume overload, graft loss
Transplant timing	Pre-emptive Post-dialysis Re-transplantation	CVD risk and comorbidity, TRAS incidence, immunological profile
Donor-related factors	Living donor/Standard criteria donor/Extended criteria donor Gender Age Comorbidities (DM, CVD, obesity)	TRAS incidence, nephron underdosing, “inherited” hypertension
Recipient-related factors	Gender Age Comorbidities (DM, CVD, obesity) Immunosuppression regimen	Arterial stiffness, CVD risk, resistant hypertension, interaction monitoring

RAS = Renin–Angiotensin System, CVD = Cardiovascular Disease, TRAS = Transplant Renal Artery Stenosis, DM = Diabetes Mellitus.

## Data Availability

No new data were created or analyzed in this study. Data sharing is not applicable to this article.
